# 111. Duration of Antibiotic Therapy after Debridement and Implant Retention in Patients with Periprosthetic Joint Infections

**DOI:** 10.1093/ofid/ofab466.111

**Published:** 2021-12-04

**Authors:** Don Bambino Geno Tai, Elie Berbari, Matthew P Abdel, Brian Lahr, Aaron J Tande

**Affiliations:** Mayo Clinic, Rochester, Minnesota

## Abstract

**Background:**

Debridement, antibiotics, and implant retention (DAIR) is appropriate for select acute postoperative and hematogenous periprosthetic joint infections (PJIs). However, the optimal duration of antimicrobial therapy in patients treated with DAIR has not been defined. Therefore, we aimed to identify the ideal duration of parenteral and oral antibiotics after DAIR.

**Methods:**

We performed a retrospective study of patients >18 years of age with hip or knee PJI managed with DAIR between January 1, 2008, and December 31, 2018, at Mayo Clinic. PJI was defined using criteria adapted from the International Consensus Meeting on PJI. The outcome was defined as either PJI recurrence or unplanned reoperation due to infection. Joint-stratified Cox proportional hazards regression models with time-dependent covariates were used to assess nonlinear effects of antibiotic duration. Hazard ratios were computed based on prespecified time points for comparison, whereas p-values represented the overall effect across the entire range of durations.

**Results:**

There were 247 unique episodes of PJI in 237 patients during the study period. Parenteral antibiotics were given in 99.2% of cases (n=245). This was followed by chronic oral antibiotic suppression in 92.2% (n=226) with a median duration of 2.2 years (1.0-4.1).

DAIR failed in 65 cases over a median follow-up of 4.4 years, with a 5-year cumulative incidence of 28.1%. After adjustment for risk factors, there was no significant association between duration of parenteral antibiotics and treatment failure (p=0.203), with no difference between four versus six weeks (HR 1.11; 95% CI 0.71-1.75) (Figure 1). However, both use and longer duration of oral antibiotic therapy was associated with a lower risk of failure (p=0.006). To account for the possibility that this association was driven by results during early follow-up, conditional analyses at one- and two-year follow-up were performed. Both showed a significantly lower risk for a longer duration of antibiotics (Figure 2).

Figure 1. Time-Dependent Analysis of Parenteral Antibiotic Duration

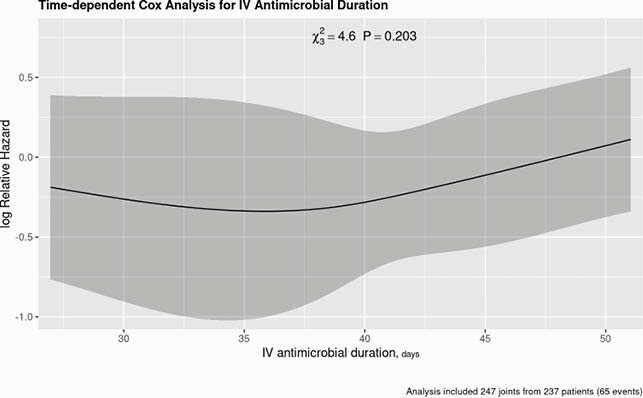

Figure 2. Time-Dependent Analysis of Oral Antibiotic Suppression Duration

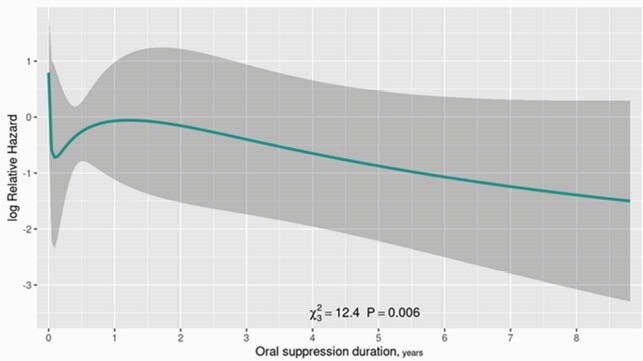

**Conclusion:**

After DAIR, efficacy from four weeks of parenteral antibiotics was no different from six weeks when followed by chronic oral antibiotic suppression. Our results could not establish an optimal duration but suggested that continuing suppression portends a lower risk of failure of DAIR.

**Disclosures:**

**Elie Berbari, MD**, **Uptodate.com** (Other Financial or Material Support, Honorary unrelated to this work) **Matthew P. Abdel, MD**, **Stryker and AAOS Board of Directors** (Board Member, Other Financial or Material Support, Royalties) **Aaron J. Tande, MD**, **UpToDate.com** (Other Financial or Material Support, Honoraria for medical writing)

